# Quantitative comparison of corneal surface areas in keratoconus and normal eyes

**DOI:** 10.1038/s41598-021-86185-3

**Published:** 2021-03-25

**Authors:** François-Xavier Crahay, Guillaume Debellemanière, Stephan Tobalem, Wassim Ghazal, Sarah Moran, Damien Gatinel

**Affiliations:** 1 Rothschild Foundation Hospital, Paris, France; 2 CHR Citadelle, Liège, Belgique

**Keywords:** Pathogenesis, Eye diseases, Corneal diseases

## Abstract

Keratoconus is a highly prevalent corneal disorder characterized by progressive corneal thinning, steepening and irregular astigmatism. To date, pathophysiology of keratoconus development and progression remains debated. In this study, we retrospectively analysed topographic elevation maps from 3227 eyes of 3227 patients (969 keratoconus and 2258 normal eyes) to calculate anterior and posterior corneal surface area. We compared results from normal eyes and keratoconus eyes using the Mann–Whitney U test. The Kruskal–Wallis test was used to compare keratoconus stages according to the Amsler–Krumeich classification. Keratoconus eyes were shown to have statistically significantly larger corneal surface areas, measured at the central 4.0 mm and 8.0 mm, and total corneal diameter. However, no significant increase in corneal surface area was seen with increasing severity of keratoconus. We suggest that these results indicate redistribution, rather than increase, of the corneal surface area with keratoconus severity.

## Introduction

Keratoconus (KC) is a bilateral asymmetric corneal condition, characterized by progressive corneal thinning and steepening, often leading to significant visual impairment^1,2^. The reported prevalence is approximately 1 in 2000^2^ and continues to rise, as advances in corneal topography and tomography allow for earlier and more widespread detection. The specific underlying cause of this condition is not yet fully understood. Many different pathways have been investigated, including biochemical, genetic, environmental and mechanical origins, and a multi-factorial origin is often cited^3^.

Keratoconus is classically defined as a bilateral non-inflammatory cone-shaped ectasia ^4,5^ of the cornea.

By definition, an ectasia refers to a dilatation or a distention of a tubular structure^2^. Keratoconus is often considered as an ectasia resulting from stromal stretching. This suggests an expansion or distention, which if true, should lead to an increased surface area. However, despite the widespread acceptance of keratoconus as an ectatic disorder, there is a lack of objective evidence to confirm corneal surface area increase in keratoconus and keratoconus progression.

The literature regarding corneal surface area in keratoconus is limited to small studies ^6–10^. Smolek and Klyce ^8^ analysed the anterior surface areas of 147 eyes using a TMS-1 videokeratograph and found no anterior cornea surface area increase in keratoconus, challenging the concept of true ectasia. More recently, Kitazawa et al., as well as Cavas-Martinez et al., found larger central corneal surface areas in keratoconus^6,7,9,10^ using data from anterior and posterior corneal surface areas obtained with an anterior segment OCT and a Sirius system corneal topographer respectively. However, it is important to note that those studies used different geometrical assumptions and algorithms for calculating the corneal surface area, and did not take the total diameter of the cornea into account. For example, Klyce and Smolek limited their analysis to anterior surface area using Placido disk reflection analysis, summing the area of individual patches along consecutive annular rings^8^, while Kitazawa assumed that the corneal surfaces were assimilated to portions of spherical caps from which discrete surface elements could be summed up.

There is no clear consensus, therefore, on the role of corneal surface area in keratoconus, as the available literature is inconclusive. To address this question, the purpose of our study was to retrospectively compare corneal surface area calculated using raw topographic elevation data, in a large dataset of 2258 normal eyes from 2258 patients, and 969 keratoconus eyes from 969 patients.

## Results

### Demographic data

Our study involved 3227 eyes of 3227 patients, of which 969 eyes had keratoconus and 2258 were normal eyes. We classified keratoconus according to the topographic criteria of the Amsler–Krumeich classification (Table 4). Characteristics of each group are summarized in Table 1.

**Table 1:** Patient demographics.

Demographic characteristics	Control	Keratoconus						
		All	p-value*	Keratoconus according the Amsler–Krumeich classification				p-value**
				AKC1	AKC2	AKC3	AKC4	
Number of eyes	2258	969		555	158	210	46	
Sex ratio (% men)	65.9	65.9	0.98	68.5	60.8	65.2	56.5	0.15
Side (% OD)	50.8	53.3	0.21	54.6	48.1	52.9	56.5	0.51
Age (years, mean ± SD)	35.2 ± 10.7	31.8 ± 11.5	< 0.0001	31.7 ± 11.1	31.9 ± 12.9	31.8 ± 11.1	32.3 ± 12.4	0.98

*OD* right eye, *SD* standard deviation, *AKC* Amsler–Krumeich classification.

*p-value of the Mann–Whitney test between control and keratoconus.

**p-value of the Kruskal–Wallis test between stages of keratoconus according to the Amsler–Krumeich Classification.

Sex ratio (65.9% and 65.9%; p = 0.98) and side (50.8% of right eyes and 53.3% of right eyes; p = 0.21) of the selected eye were similar in both groups. However, keratoconus patients were slightly younger than the control group (31.8 and 35.2 years, respectively; p < 0.0001). No difference was observed between keratoconus subgroups.

### Intergroup comparison of topographic parameters

**Figure 1:**
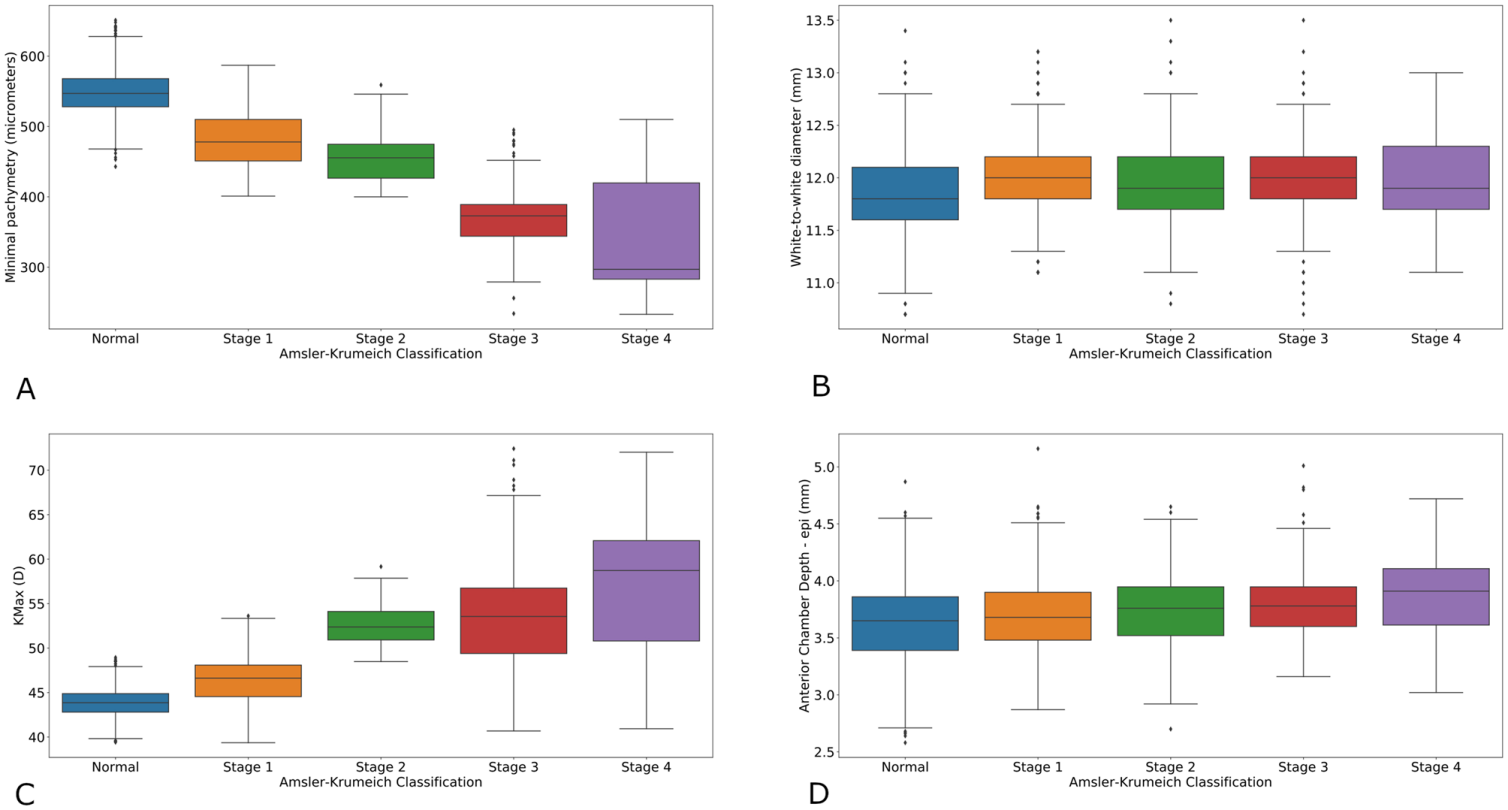
(**A**) Minimal pachymetry (micrometer) in normal eyes and keratoconus stages according to the Amsler–Krumeich Classification. Box-plot. (**B**) White to white corneal diameter (millimeter) in normal eyes and keratoconus stages according to the Amsler–Krumeich classification. Box plot. (**C**) Maximal keratometry (Kmax, dioptre) in normal eyes and keratoconus stages according to the Amsler–Krumeich Classification. Box-Plot. *Kmax* maximal keratometry, *D* dioptre, (**D**) Anterior chamber depth from epithelium (millimeter) in normal eyes and keratoconus stages according to the Amsler–Krumeich Classification. Box-Plot.

Minimal pachymetry was significantly thinner in keratoconus eyes than control (445.4 µm and 548.7 µm; p < 0.0001) and corneal diameter was larger in keratoconus eyes compared to normal eyes (12.01 mm and 11.82 mm; p < 0.0001). Maximal keratometry was statistically significantly steeper in the keratoconus group (49.57 and 43.87; p < 0.0001). Mean anterior keratometry values in the centered 3.0 mm zone, in the 3–5 mm ring were statistically steeper in keratoconus eyes (p < 0.0001). This data is summarized in Table 2 and in Fig. 1A–D.

**Table 2:** Mean topographic and keratometry parameters for control and keratoconus groups.

Topographic parameters: mean ± SD	Control	Keratoconus							
		All	p-value*	Percentage of difference*** (%)	AKC1	AKC2	AKC3	AKC4	p-value**
Minimal pachymetry (µm ± SD)	549 ± 30	445 ± 65	< 0.0001	-19	480 ± 41	456 ± 36	369 ± 40	341 ± 81	< 0.0001
Corneal diameter (mm ± SD)	11.82 ± 0.35	12.01 ± 0.39	< 0.0001	+ 2	12.03 ± 0.37	11.94 ± 0.44	11.99 ± 0.41	11.95 ± 0.44	0.0069
Maximal keratometry (D ± SD)	43.87 ± 1.53	49.69 ± 5.47	< 0.0001	+ 13	46.44 ± 2.46	52.65 ± 2.14	53.84 ± 5.84	57.30 ± 7.67	< 0.0001
Anterior chamber depth from epithelium (mm ± SD)	3.62 ± 0.33	3.74 ± 0.33	< 0.0001	+ 3.3	3.70 ± 0.33	3.75 ± 0.33	3.81 ± 0.30	3.86 ± 0.37	< 0.0001
Mean anterior keratometry 0–3 mm (D ± SD)	43.28 ± 1.47	46.67 ± 3.32	< 0.0001	+ 8	44.77 ± 1.70	48.48 ± 1.06	49.20 ± 3.62	51.69 ± 4.98	< 0.0001
Mean anterior keratometry 3–5 mm (D ± SD)	43.01 ± 1.45	44.87 ± 2. 31	< 0.0001	+ 4	43.78 ± 1.46	45.82 ± 1.34	46.21 ± 2.56	47.64 ± 3.37	< 0.0001

*D* dioptre, *SD* standard deviation.

*p-value of the Mann–Whitney test between control and keratoconus.

**p-value of the Kruskal–Wallis test between stages of keratoconus according to the Amsler–Krumeich Classification.

***Percentage of difference between means of keratoconus and control groups.

In keratoconus subgroups, keratometry was significantly steeper and pachymetry thinner with keratoconus severity. (p < 0.0001).

Anterior chamber depth also increased with keratoconus severity (p < 0.001). Moreover, there was a small, but significant difference in corneal diameter between keratoconus subgroups, without any clear trend.

### Corneal surface areas

**Figure 2:**
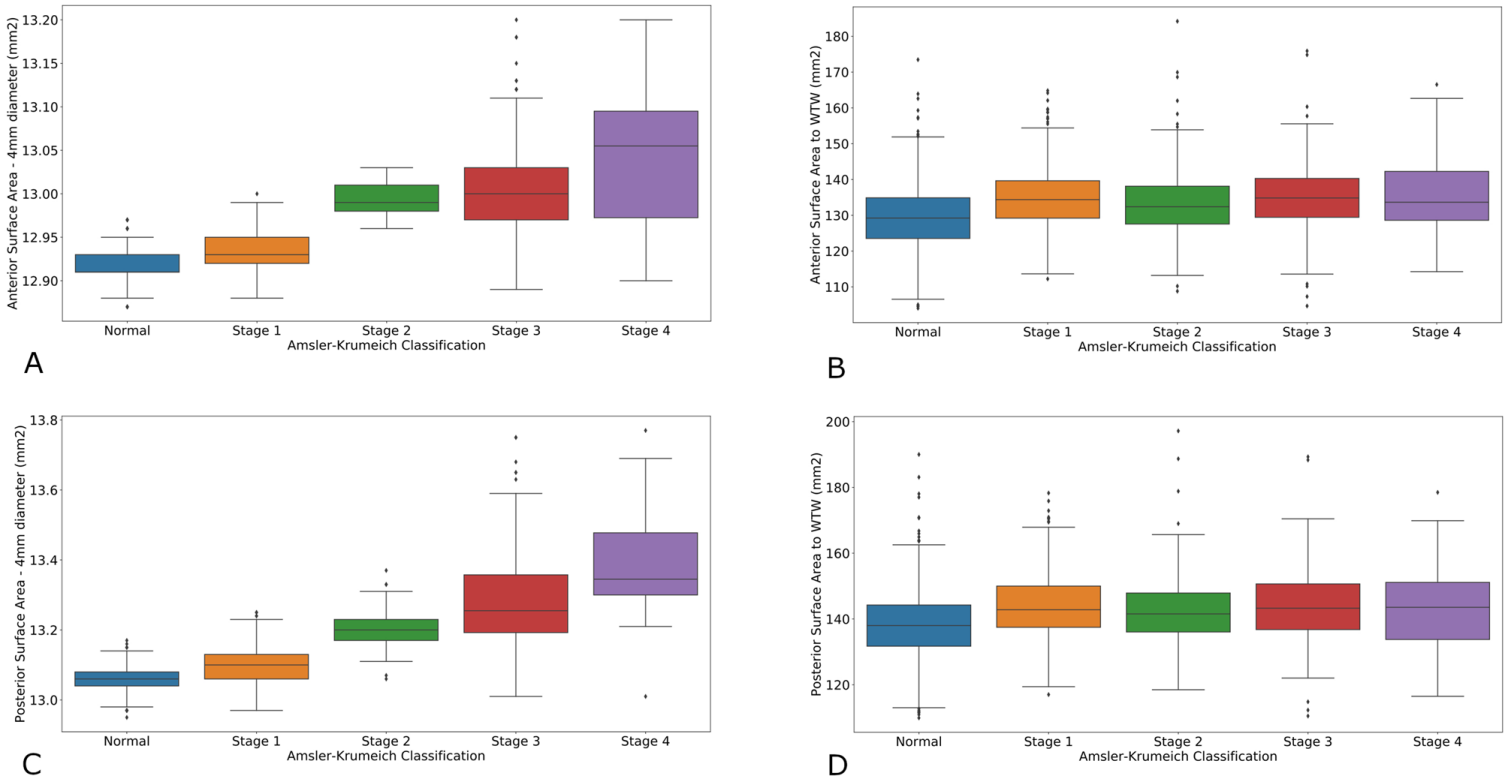
(**A**) Anterior corneal surface area measured at central 4.0 mm diameter (square millimeter) in normal eyes and keratoconus stages according to the Amsler–Krumeich Classification. Box-Plot. (**B**) Total anterior corneal surface area (square millimeter) in normal eyes and keratoconus stages according to the Amsler–Krumeich Classification. Box-Plot. *WTW* total corneal diameter. (**C**) Posterior corneal surface area measured at central 4.0 mm diameter (square millimeter) in normal eyes and keratoconus stages according to the Amsler–Krumeich Classification. Box-Plot. (**D**) Total posterior corneal surface area (square millimeter) in normal eyes and keratoconus stages according to the Amsler–Krumeich Classification. Box-Plot. *WTW* total corneal diameter.

Anterior and posterior surface areas were calculated at the central 4.0 mm and 8.0 mm diameter from raw elevation data. Anterior and posterior surfaces areas from 8.0 mm to the limbus were extrapolated using a best fitting curve method. Distributions showed wide overlap between normal and keratoconus values. Data is summarized in Table 3. Distribution of corneal surface areas is represented in Fig. 2A–D.

**Table 3:** Mean anterior and posterior corneal surface areas measured at central 4.0 mm, 8.0 mm and at the measured corneal diameter.

Corneal surface area: mean ± SD (mm^2^)	Control	Keratoconus							
		All	p-value*	Percentage of difference*** (%)	AKC1	AKC2	AKC3	AKC4	p-value**
**Anterior surface area : mean ± SD (mm**^**2**^**)**									
Central 4 mm	12.92 ± 0.02	12.96 ± 0.05	< 0.0001	+ 0.4	12.93 ± 0.02	12.99 ± 0.02	13.00 ± 0.05	13.04 ± 0.075	< 0.0001
Central 8 mm	54.99 ± 0.30	55.31 ± 0.46	< 0.0001	+ 0.6	55.10 ± 0.30	55.50 ± 0.29	55.58 ± 0.52	55.90 ± 0.72	< 0.0001
Entire corneal surface area****	129.3 ± 8.18	134.7 ± 9.63	< 0.0001	+ 4.1	134.9 ± 8.84	133.7 ± 11.02	135.1 ± 10.14	135.3 ± 11.32	0.1669
**Posterior surface area: mean ± SD (mm**^**2**^**)**									
Central 4 mm	13.06 ± 0.03	13.17 ± 0.12	< 0.0001	+ 0.8	13.10 ± 0.05	13.20 ± 0.05	13.28 ± 0.14	13.39 ± 0.14	< 0.0001
Central 8 mm	57.09 ± 0.62	57.65 ± 0.89	< 0.0001	+ 1	57.25 ± 0.60	57.92 ± 0.65	58.23 ± 0.96	58.90 ± 1.27	< 0.0001
Entire corneal surface area****	138.1 ± 9.24	143.8 ± 10.84	< 0.001	+ 4.1	144.0 ± 10.05	142.8 ± 12.15	143.9 ± 11.43	144.0 ± 12.53	0.3348

*p-value of the Mann–Whitney test between control and keratoconus.

**p-value of the Kruskal–Wallis test between stages of keratoconus according to the Amsler–Krumeich Classification.

***Percentage of difference between means of keratoconus and control groups.

****Total corneal surface area are extrapolated up to the measured corneal diameter from data available from central 1.0–8.0 mm diameter using a best fitting curve method.

Anterior corneal surface area measured at the central 4.0 mm and 8.0 mm diameter area were significantly larger in keratoconus (12.96 mm^2^ and 55.31 mm^2^, respectively) compared to normal eyes (12.92 mm^2^ and 54.99 mm^2^, respectively). Central surface area increased with keratoconus severity, according to the Amsler–Krumeich classification (Fig. 2A).

Total anterior corneal surface area calculated up to the measured corneal diameter was significantly larger in keratoconus (134.74 mm^2^) than in controls (129.3 mm^2^). However, total anterior corneal surface area showed no significant difference between keratoconus subgroups according to the Amsler–Krumeich classification (p = 0.1669) (Fig. 2B).

Posterior corneal surface area measured at the central 4.0 mm and 8.0 mm diameter area were also significantly larger in keratoconus, and increased with keratoconus severity (Fig. 2C).

Total posterior corneal surface area calculated up to corneal diameter was larger in keratoconus compared to normal eyes (143.8mm^2^ and 138.1 mm^2^, respectively) but showed no significant difference between groups of the Amsler–Krumeich Classification (p = 0.334) (Fig. 2D).

## Discussion

In order to calculate corneal surface areas, we used a geometrical method inspired from landscape measurements^11^ using raw elevation data, and no geometrical assumption on the global shape or local curvature of the measured corneal surface. Elevation data was obtained from topographic measurements using the Orbscan II, which have been shown to be repeatable^12^. Such a method of dividing each discrete point into eight 3D triangles, is commonly used to calculate surface area ^11^. We deduced the surface area from 8.0 mm to the measured corneal diameter for each cornea independently, by plotting the surface area for each ring diameter (from central 1.0 mm to central 8.0 mm diameter) and by fitting a polynomial of degree 3 to the available points. Sex-ratio between each group was adjusted in order to eliminate a possible sex-induced bias.

Keratometry of eyes with keratoconus were steeper, but this difference decreased with the diameter of analysis. In our opinion, this could corroborate a curvature redistribution induced by the hyperprolate corneal profile of corneas with keratoconus^13^.

Keratoconus anterior and posterior surface areas were greater than control. However, there were wide overlaps in the distribution of values between normal eyes and keratoconus. This emphasizes that, in terms of corneal surface area measurements, no clear cut-off value can be set to discriminate normal corneas from keratoconus. The differences in surface area (0.4–4.1%) between the two groups however, are notably less than the differences observed for maximal keratometry and minimal pachymetry (13% and 19% respectively) and although statistically significant, in our view, are unlikely to be clinically relevant.

Furthermore, although the central 4.0 mm and 8.0 mm diameter corneal surface area increased with keratoconus severity, total corneal surface area did not. When analysed on the largest available zone, average keratometry and surface area values were relatively conserved suggesting a redistribution rather than a net increase. This observation is consistent with the Gauss's ***Theorema Egregium*** (Latin for "Remarkable Theorem") that states that the Gaussian curvature, which at a point is the product of the principal curvatures, does not change if one bends the surface without stretching it. Some authors have proposed that the biomechanical modification encountered in keratoconus corneas is focal in nature, rather than a uniform generalized weakening ^14^. They proposed the concept that a focal reduction in the elastic modulus precipitates a cycle of biomechanical decompensation, driven by asymmetry in the biomechanical properties. A repeating cycle of increased strain and stress redistribution would then lead to subsequent focal steepening and thinning ^15^.

As keratoconus progresses, the central steepening is associated with peripheral flattening, which is consistent with a central increase in surface area and a peripheral relative reduction. The increasing amount of flattening in thicker peripheral corneal regions should allow higher stresses to be tolerated without requiring any accompanying increase in surface area. This surface redistribution matched the curvature redistribution in our study and is reflected in the increased prolateness of keratoconus corneas.

The observed difference in surface area between control and keratoconus eyes may be explained by the larger corneal diameter and the deeper anterior chamber in keratoconus. Indeed, the entire corneal surface area is largely influenced by the corneal diameter and possibly by the anterior chamber depth. In fact, if we make an analogy between the cornea and a spherical cap, the corneal diameter and the anterior chamber depth intervene according to the theorical formula:

$$Corneal surface area = \pi \times \left( {\left( {\frac{spherical cap diameter}{2}} \right)^{2} + \left( {height of spherical cap} \right)^{2} } \right) \approx \pi \times \left( {\left( {\frac{corneal diameter}{2}} \right)^{2} + \left( { \approx anterior chamber depth} \right)^{2} } \right)$$

This formula emphases the relative importance of the square of corneal diameter and anterior chamber depth in surface area calculation.

Our study demonstrates an increase in corneal surface area in eyes with keratoconus, although the total corneal surface area remains constant with increasing keratoconus severity. We hypothesize that corneal surface area, as well as curvature, is redistributed without significant corneal stretching. As such, this suggests that keratoconus is a permanent corneal deformation with a stable total surface area, and may be considered as an extreme form of corneal warpage caused by structural damage secondary to stromal degeneration and external forces^8^.

Otherwise, when compared to control eyes, keratoconus eyes showed increased corneal surface area, but also increased corneal diameter and anterior chamber depth. As of yet, it is unclear whether these increased values are a sign of keratoconus or may represent potential risk factors.

Further studies are required to improve our understanding of the role of corneal diameter and corneal surface area in the development of keratoconus..

## Methods

### Patients

This retrospective study included subjects examined at the Department of Anterior Segment and Refractive surgery at Rothschild Foundation Hospital, Paris, France. The study and data collection were achieved with approval from the Rothschild Foundation Institutional Review Board which followed the tenets of the Declaration of Helsinki. Informed consent was obtained from all subjects prior to their participation in this study.

Inclusion criteria for the control group were healthy eyes that did not meet any exclusion criteria and were not diagnosed with keratoconus.

Keratoconus was diagnosed mainly on the basis of associated characteristic topographic patterns and keratometry. Topographic characteristics included an asymmetric bowtie pattern in corneal topography, a central keratometry superior to 47.2 dioptres, pathological anterior or posterior corneal elevation. Eyes with forme fruste keratoconus were included.

Exclusion criteria in both groups were any previous ocular surgery (e.g. penetrating keratoplasty, lamellar keratoplasty, corneal rings, corneal collagen cross-linking) and any other ocular disease. Patients using rigid gas permeable contact lenses were also excluded. Patients using soft contact lenses were asked to stop wearing them two weeks before the examination. Topographies with missing data within the central 8 mm were excluded.

First, elevation maps obtained by Orbscan II at Rothschild Foundation Hospital were classified using a machine learning algorithm^16^ as keratoconus or normal. We obtained 12 587 elevation maps from 8162 healthy eyes and 7003 elevation maps from 2041 keratoconus. Topographies were manually checked for inclusion and exclusion criteria.

If more than one topography was available for the same eye, only one map was randomly kept to obtain 6863 elevation maps from 3730 normal patients and 2236 maps from 969 keratoconus patients.

A single elevation map from one eye of each patient was then randomly selected, leaving 3730 maps from 3730 patients and 969 maps from 969 patients.

1472 normal elevation maps from female patients were then randomly removed in order to equalize the sex-ratio in both groups, and eliminate a potential bias.

**Figure 3:**
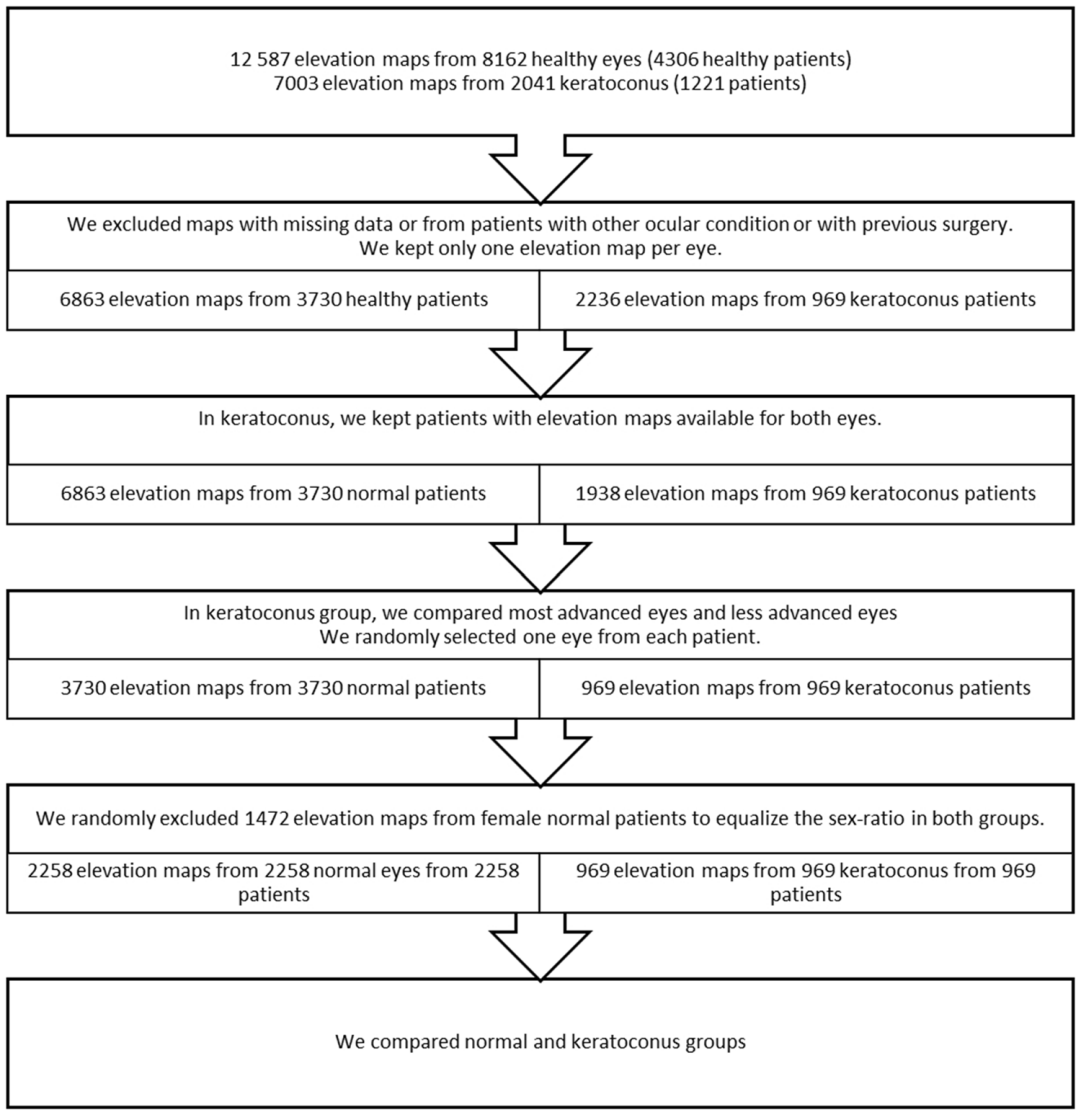
Flow diagram of patient selection process.

Figure 3 summarizes the process to select the elevation maps studied.

Keratoconus was classified following the Amsler–Krumeich classification ^17^. Only topographic criteria were used. Grades are summarized in Table 4.

**Table 4:** Amsler–Krumeich classification.

Grade	Characteristics
**Adapted Amsler–Krumeich classification**	
Stage 1	Mean central keratometry < 48 D
Stage 2	Mean central keratometry from < 53 D Minimum corneal thickness > 400 µm
Stage 3	Mean central keratometry from > 53 D Minimum corneal thickness from 200 to 400 µm
Stage 4	Mean central keratometry > 55 D Minimum corneal thickness > 200 µm

We only used topographic criteria, i.e. mean central keratometry readings and minimum corneal thickness.

### Corneal topography method

Topography was obtained using the Orbscan II corneal topographer (Bausch & Lomb). This device uses a Placido disk with 40 rings combined with 40 scans of slit lamp acquisition. Scans were obtained by qualified technicians using standard protocol for Orbscan topography acquisition. Poor quality exams were repeated. The Orbscan’s software provided anterior and posterior elevation maps including 10,000 measurements for a corneal zone of 10 by 10 mm. The elevation maps were calculated from a reference float best fit sphere^18^.

Orbscan II also provided biometric and keratometry values: corneal diameter (horizontal white to white), minimal pachymetry, maximal keratometry, and cornea irregularities in the central 3.0 mm diameter and the 3–5 mm ring.

All topographies were acquired at Rothschild Foundation Hospital. Data was exported from Orbscan and analysed and classified using NumPy v1.18.0 package.

### Corneal surface calculation

**Figure 4:**
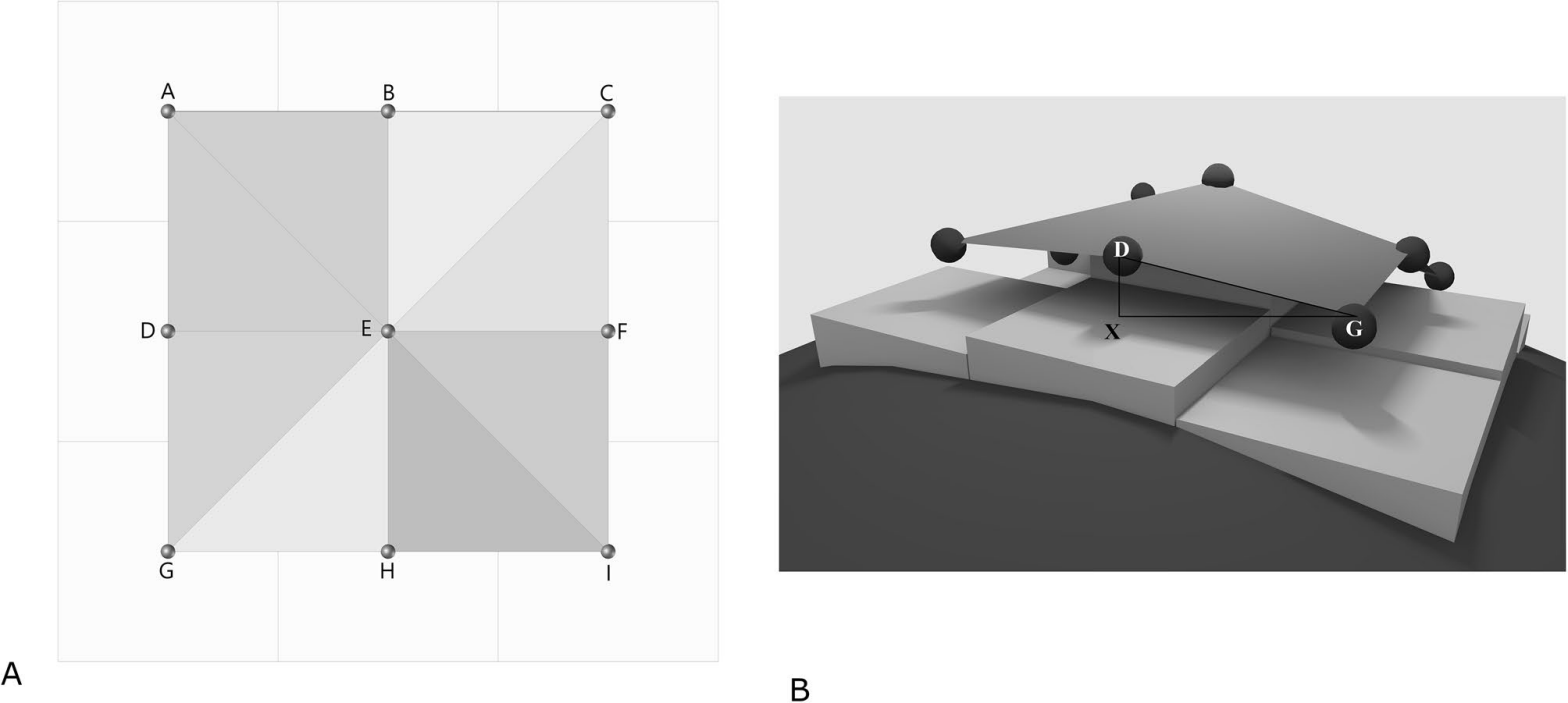
(**A**) Diagram representing the elevation of 8 points adjacent to the measured point. Each point represents the center of a square cell whose side length is equal to half the distance between each measured point. We are trying to evaluate the surface of this cell, which depends on the elevation of the central point, and the 8 points which are adjacent to it. There are 8 triangles that meet at the measured point. (**B**) The lengths of the sides of each triangles are calculated using the Pythagorean theorem. The distance between two elevation points is calculated using the formula ${\text{DG}} = \sqrt {\left( {{\text{DX}}^{{2}} + {\text{XG}}^{{2}} } \right)}$ where DX is the difference of elevation values and XG the distance between two measured points. Then using Euclid’s theorem of similar triangles, the length values are divided by two to fit the area surrounding the central point (E). The surface area of each triangle is calculated using those half lengths. Finally, the sum of the 8 triangle area values corresponds to the area of the cell surrounding E.

Corneal surface area was calculated from anterior and posterior elevation raw data using a method previously described for landscape surface area calculation^11^. The corneal surface area around each point of the elevation map is calculated using the elevation value of this point and of the 8 surrounding points. The point of interest is connected to the 8 surrounding points, generating 8 triangles with different orientation in space. The vertices of each triangle have differing elevations. The lengths for the sides of the 8 triangles can be calculated using the Pythagorean Theorem, incorporating slope because of each vertex’s unique elevation value. All length values are divided by 2, to consider only the portion of triangles that fall within the central point’s boundary. The area of the triangles that lie in the central cell boundaries was then calculated from the elevation values, using the Pythagorean theorem and Euclid theorem (Fig. 4). The surface area of each point is equal to the sum of the 8 triangle areas.

Using this method, the corneal area is gradually calculated for concentric areas ranging from the central 1.0 mm to the central 8.0 mm diameter.

Missing elevation data from 8.0 mm diameter to the limbus did not allow corneal surface area calculation. Thus we extrapolated corneal surface area from the central 8.0 mm to the measured total corneal diameter by plotting the surface area for each ring diameter (from central 1.0 mm to central 8.0 mm diameter) and using a least square polynomial fit of degree 3. These calculations were achieved using Python 3.7 and the NumPy v1.18.0 package.

### Data analysis

Data was compiled in Microsoft Excel files. Variance analysis and descriptive statistics were performed with Microsoft Excel and Prism GraphPad software. Normality tests were performed for each parameter using the D’agostino-Pearson normality test. Since the data was not normally distributed, we used non-parametric Mann–Whitney U test to compare parameters of normal eyes and keratoconus. To compare different stages of keracotonus, we used the Krukal-Wallis test.

A *P value of* less than 0.05 was considered statistically significant.

## References

[1] Shajari M (2019). Evaluation of keratoconus progression. Br. J. Ophthalmol..

[2] Group of Panelists for the Global Delphi Panel of Keratoconus and Ectatic Diseases *et al.* Global Consensus on Keratoconus and Ectatic Diseases. *Cornea* **34**, 359–369 (2015). 10.1097/ICO.0000000000000408 25738235

[3] Sugar J, Macsai MS (2012). What causes keratoconus?. Cornea.

[4] Yaron S (1998). Rabinowitz. Keratoconus. Surv. Ophthalmol..

[5] Krachmer JH, Feder RS, Belin MW (1984). Keratoconus and related noninflammatory corneal thinning disorders. Surv. Ophthalmol..

[6] Cavas-Martínez, F. *et al.* Geometrical custom modeling of human cornea in vivo and its use for the diagnosis of corneal ectasia. *PLoS One* **9**, (2014). 10.1371/journal.pone.0110249 PMC4201525 25329896

[7] Itoi, M. *et al.* Anterior and posterior ratio of corneal surface areas: A novel index for detecting early stage keratoconus. *PLoS One* **15**, (2020). 10.1371/journal.pone.0231074 PMC7117727 32240243

[8] Smolek, M. K. & Klyce, S. D. Is Keratoconus a True Ectasia? **118**, (2000). 10.1001/archopht.118.9.1179 10980762

[9] Cavas-Martínez, F., Bataille, L., Fernández-Pacheco, D. G., Cañavate, F. J. F. & Alió, J. L. A new approach to keratoconus detection based on corneal morphogeometric analysis. *PLoS One* **12**, (2017). 10.1371/journal.pone.0184569 PMC5590974 28886157

[10] Kitazawa K (2018). Involvement of anterior and posterior corneal surface area imbalance in the pathological change of keratoconus. Sci. Rep..

[11] Jenness JS (2005). Calculating landscape surface area from digital elevation models. Wildl. Soc. Bull..

[12] Guilbert E (2016). Repeatability of keratometry measurements obtained with three topographers in keratoconic and normal corneas. J. Refract. Surg..

[13] Schlegel Z, Hoang-Xuan T, Gatinel D (2008). Comparison of and correlation between anterior and posterior corneal elevation maps in normal eyes and keratoconus-suspect eyes. J. Cataract Refract. Surg..

[14] Roberts CJ, Dupps WJ (2014). Biomechanics of corneal ectasia and biomechanical treatments. J. Cataract Refract. Surg..

[15] Gatinel D (2018). Challenging the “No Rub, No Cone” Keratoconus Conjecture. Int. J. Keratoconus Ectatic Corneal Dis..

[16] Zéboulon P, Debellemanière G, Bouvet M, Gatinel D (2020). Corneal topography raw data classification using a convolutional neural network. Am. J. Ophthalmol..

[17] Krumeich JH, Daniel J, Knülle A (1998). Live-epikeratophakia for keratoconus. J. Cataract Refract. Surg..

[18] Cairns G, McGhee CNJ (2005). Orbscan computerized topography: Attributes, applications, and limitations. J. Cataract Refract. Surg..

